# Physiotherapy using a free-standing robotic exoskeleton for patients with spinal cord injury: a feasibility study

**DOI:** 10.1186/s12984-021-00967-4

**Published:** 2021-12-25

**Authors:** Nicola Postol, Neil J. Spratt, Andrew Bivard, Jodie Marquez

**Affiliations:** 1 University of Newcastle, Callaghan, Australia; 2 Hunter Medical Research Institute, New Lambton Heights, Australia; 3 Hunter New England Local Health District, New South Wales, Australia; 4 University of Melbourne, Melbourne, Australia

**Keywords:** Robotic exoskeleton, Free-standing, Spinal cord injury, Neuro-rehabilitation, Feasibility

## Abstract

**Background:**

Evidence is emerging for the use of overground lower limb robotic exoskeletons in the rehabilitation of people with spinal cord injury (SCI), with suggested benefits for gait speed, bladder and bowel function, pain management and spasticity. To date, research has focused on devices that require the user to support themselves with a walking aid. This often precludes use by those with severe trunk, postural or upper limb deficits and places the user in a suboptimal, flexed standing position. Free-standing exoskeletons enable people with higher level injuries to exercise in an upright position. This study aimed to evaluate the feasibility of therapy with a free-standing exoskeleton for those with SCI, and to determine the potential health-related benefits of this intervention.

**Methods:**

This 12-week intervention study with 12-week waitlist control and 12-week follow up, provided people with SCI scoring < 5 on the mobility section of the spinal cord independence measure (SCIM-III) twice weekly therapy in the REX (Rex Bionics, Auckland, NZ), a free-standing lower limb robotic exoskeleton. The primary outcome measure of interest was function, as measured on the SCIM-III. A battery of secondary outcomes was included. Participants also completed a survey on their perceptions of this treatment modality, to determine acceptability.

**Results:**

Forty-one potential participants were screened for eligibility. Two females (one ASIA A, one ASIA C) and one male (ASIA B) completed all 24 intervention sessions, and the follow up assessment. One participant showed positive trends in function, fatigue, quality of life and mood during the intervention phase. Grip and quadriceps strength, and lower limb motor function improved in another. Two improved their percentage of lean body mass during the intervention phase. Remaining results were varied across patients, time points and outcomes. The intervention was highly acceptable to all participants.

**Conclusion:**

With three of 41 potential participants being eligible and completing this study, our results show that there are potential benefits of exercise in a free-standing exoskeleton for people with severe mobility impairment due to SCI, for a small subset of patients. Further research is warranted to determine those most likely to benefit, and the type of benefit depending on the patient characteristics.

*Trial registration* The trial was registered prospectively on 20 April 2018 at www.anzctr.org.au/ (ACTRN12618000626268)

**Supplementary Information:**

The online version contains supplementary material available at 10.1186/s12984-021-00967-4.

## Background

In Australia there are 12,000 people living with spinal cord injury (SCI), with 350–400 new cases per year [1]. Those with SCI have varying degrees of ability depending on the level and nature of the injury. It is reported that 60% of people with SCI are reliant on a wheelchair for mobility [2], and many of those require significant physical assistance to be able to access exercise in standing. Weight-bearing exercises such as sit to stand and locomotor training are essential functional components of therapy, necessary to strengthen or form new neural pathways and cortical adaptations [3], and maximise recovery in those with SCI [3, 4]. Prolonged wheelchair use is known to lead to decreased bone mass and muscle mass and increased fat mass [5, 6]. Weight-bearing exercise is important in the management of some of the secondary complications of SCI, such as decreased bone density, and bladder and bowel function [7].

Robotic exoskeletons are wearable devices which have powered joints and assist with movement and mobility [8]. The intended purpose of these devices can be separated into two categories: rehabilitation, or gait assistance [9]. There are currently numerous devices on the market, however research into the use of overground robotic exoskeletons in the rehabilitation of those with SCI has focused on three devices: ReWalk [10], Ekso [11] and Indego [12], all of which primarily focus on assisting gait to facilitate ambulation. All three devices have actuated hip and knee joints, but unpowered ankle joints, and require the user to support their weight with a walking aid, such as crutches or a walking frame. Clinical feasibility of these devices has been demonstrated [7, 13–16], and research into clinical benefits suggests possible improvements in bladder and bowel function [14, 15, 17–19], pain [14, 15, 17, 20], spasticity [14, 15, 20, 21], bone density [22], lean body mass [23], muscle tone [6, 15, 17], and improved walking speed within the device [13, 14]. Improvements in mood and mental state have also been reported [14]. Most studies report no adverse events [13, 22, 24], although there have been documented incidences of bruising on participants’ lower limbs [25], and two incidents of lower limb fractures [26].

Despite the reported benefits, limitations of therapy using exoskeletons which require the user to use their upper limbs for support have been raised [27–29]. Therapists, with experience in using the devices clinically, have reported that devices with self-balancing capabilities, and powered ankle joints, may provide more benefit to people with SCI [27]. As a large proportion of those with SCI have tetraplegia, the ability to access weight-bearing exercise without the need to rely on using upper limbs to support themselves, is essential [9, 27]. Furthermore, a 2020 study by Smith et al. suggested that long term use of crutches with an exoskeleton may pose greater risk of injury to the upper extremities for a person with SCI, due to increased forces compared with crutch use alone [30]. The Rex Bionics (Auckland, NZ) lower limb robotic exoskeleton, REX [31], is currently the only commercially available free-standing device, which facilitates exercise in standing, without the use of a walking aid. Therapy with the REX may therefore offer distinct advantages to other exoskeletons, with its free-standing capacity, actuated ankles, and focus on rehabilitation exercises rather than use as a gait assist device.

Although these advantages have been postulated, there is very limited available evidence to support or refute the benefits of free-standing robotic exoskeletons. A 2017 study of 20 people with SCI found that it was feasible and safe to transfer in and out of a free-standing exoskeleton, and complete one session of exercise with it [16]. No study has evaluated the benefits of free-standing robotic exoskeletons as a rehabilitation tool for people with SCI, and in light of their unique design features, further investigation is warranted. We aimed to evaluate the feasibility of using a free-standing exoskeleton for a course of exercise therapy, for people with severe mobility impairment as a result of SCI. More specifically, we aimed to assess the study procedures for their acceptability, to estimate likely rates of recruitment and retention of subjects, and to determine any health-related benefits in order to guide the development of a future powered trial.

## Method

### Study design

This study was originally planned as a cohort study. However, due to a lack of eligible participants, this work is being presented as a feasibility study. This was a pre-post interventional trial with a 12-week waitlist control and 12-week follow-up assessment. This study received approval from the Hunter New England Human Research Ethics Committee and was co-registered with the University of Newcastle Ethics Committee. The study was registered with the Australia New Zealand Clinical Trials Registry (ACTRN12618000626268).

### Participants

The research was promoted via public forums using social, print and news media. In addition, clinicians working in the Spinal Cord Injury Service (Hunter New England Health) provided information to clients about the research.

Potential participants attended a screening assessment to determine eligibility. This involved a medical screen, cognitive assessment and body measurements to determine ability to comply with the therapy and meet sizing requirements of the robotic device. Criteria for inclusion were (1) diagnosis of traumatic or non-traumatic SCI at least 3 months prior to enrolment, (2) resident of the Hunter region, (3) 18 years of age or older, (4) discharged from inpatient rehabilitation, (5) severe mobility impairment and reliant on wheelchair, mobility aid, or the assistance of others for standing activities (score < 5 on items 12–14 of the mobility section of the spinal cord independence measure (SCIM-III) [32]). Exclusion criteria included: (1) weight > 100 kg or < 40 kg, or height > 6′4″ or < 4′8″ (as per the recommendations of the robotic manufacturer), (2) pregnancy, (3) unstable or severe cardiac or respiratory compromise, (4) non-consolidated fractures in lower limbs/pelvis/spine or diagnosed severe osteopenia (t-score ≤ −2.5), (5) significant cognitive impairment (Montreal Cognitive Assessment (MoCA [33]) score of < 19), (6) any medical condition which limits the ability to exercise in an upright position or (7) a history of pathological fractures in the lower limbs in the last 2 years.

### Device

The Rex Bionics (Auckland, NZ) lower limb robotic exoskeleton, REX, is registered with Therapeutic Goods Administration of Australia as a class one medical device approved for use in clinical settings under the supervision of a therapist trained in its use. This free-standing device enables standing in a fully upright, weight-bearing position, without the use of crutches. It has 10 linear actuators (two each in the hip and ankle, and one in the knee, in each leg) [31] and therefore facilitates movement of all lower limb joints. The device can perform various functional exercises in addition to gait training. As the device does not have any biofeedback mechanism, there is no adjustment of movement regardless of participant capability. The REX moves at a speed of 0.5 m/s [34] and is controlled by a joystick on the right arm of the device, which is therapist operated.

Where possible, transfers into the device were done through standing with assistance from the therapist. Where this could not be achieved, a sling lifter was used. Participant transfers into and out of the exoskeleton were always performed with it positioned in sitting. Participants’ measurements were taken at their initial assessment and the device thigh, shin and foot length adjusted accordingly before each session. The built-in pelvic harness provided additional support.

### Intervention

The intervention involved two sessions of exercise therapy facilitated by the exoskeleton per week for 12 weeks. Each session consisted of up to half an hour of individualised therapy, prescribed and administered by a Rex Bionics accredited physiotherapist. This included upright weight-bearing exercise within the device as tolerated by the participant and was a combination of sit to stand practice, standing tolerance, weight shift, trunk control exercises, stepping practice, side stepping, squats, upper limb exercises and gait practice, individually tailored to meet the abilities and needs of the client.

Interventions were progressed according to the individual participants’ abilities as deemed appropriate by the administering physiotherapist. Interventions were modified or ceased if, in communication with the participant, the researchers deemed that this was necessary. Participants were encouraged to continue with ongoing home exercise programs as per their regular physiotherapy instruction.

### Outcome measures

At the initial appointment, assessments were conducted to establish baseline and demographic data which included injury classification according to the International Standards for Neurological Classification of SCI (ASIA scale) [35]. Participants were then assigned to a 12-week waitlist. The primary outcome of interest was functional ability as measured by the SCIM-III which is scored out of 100, with 100 indicating full function. The SCIM-III is validated in both traumatic and non-traumatic SCI populations [32, 36], and measures all aspects of physical function including mobility, bladder and bowel, feeding and respiratory function.

Due to the absence of previous research on this subject, we used a battery of secondary outcome measures to evaluate a range of potential therapeutic effects. The lower extremity motor scale (LEMS) [37] is scored out of 50, with 50 indicating normal strength in hip flexors, knee extensors, ankle dorsiflexors, long toe extensors and ankle plantarflexors. The Tardieu scale [38] was used to evaluate spasticity in the quadriceps, hamstrings and gastrocnemius muscles. The time taken to complete the five times sit to stand test (FTSST) [39] was measured, with a shorter time indicating higher function. The functional reach test (FR) [40] was used to measure static balance. Bioelectrical impedance analysis (BIA), which measures the percentage of lean body mass, was evaluated using the Biodynamics BIA 450 bioimpedance analyser (Washington state, USA) [41]. Quadriceps and grip strength were measured in kilograms of force using dynamometers with the combined average of both sides reported. The hospital anxiety and depression scale (HADS) [42] is scored out of 42, with half of the questions related to each of the two domains, a score of zero indicating no anxiety or depression. The fatigue assessment scale (FAS) [43], which is out of 50, indicates no fatigue with a score of zero. The health-related quality of life (QoL) assessment (short form 8—SF8) suggests maximum QoL with a score of zero out of 42 [44].

Participants’ perceptions of the intervention were assessed via a survey developed by this research team (please see Additional file 1), which contained 18 questions in total. The questions covered five domains related to safety (three questions), likeability (four questions), comfort (five questions), useability (three questions), and desire to continue using the device (one question). These 16 closed questions were each scored out of five, giving a maximum total possible score of 80, indicating positivity about the intervention. Two open questions asked for participant views on the most and least liked features of the intervention. Adverse events occurring during the therapy sessions and throughout the duration of the program, including non-compliance/drop-outs were also recorded and a participation log was kept by the therapist.

The outcome measures were administered upon enrolment (week 0), prior to the commencement of the intervention (week 12), mid-way through the intervention phase (week 18), at the end of the 12-week intervention (week 24), and then again 12 weeks after the intervention had been completed (week 36). The wait phase was used to determine if the participants were clinically stable prior to the intervention. A mid-intervention analysis was used to establish if there were any treatment effects with the shorter duration, and the follow up analysis enabled us to determine if there was a lasting treatment effect in any outcomes.

### Data analysis

Changes at each time point across the study duration, for each individual, were observed for each of the outcomes and the data were presented graphically to observe for trends. Data is presented for each individual participant.

## Results

### Participants

**Fig. 1:**
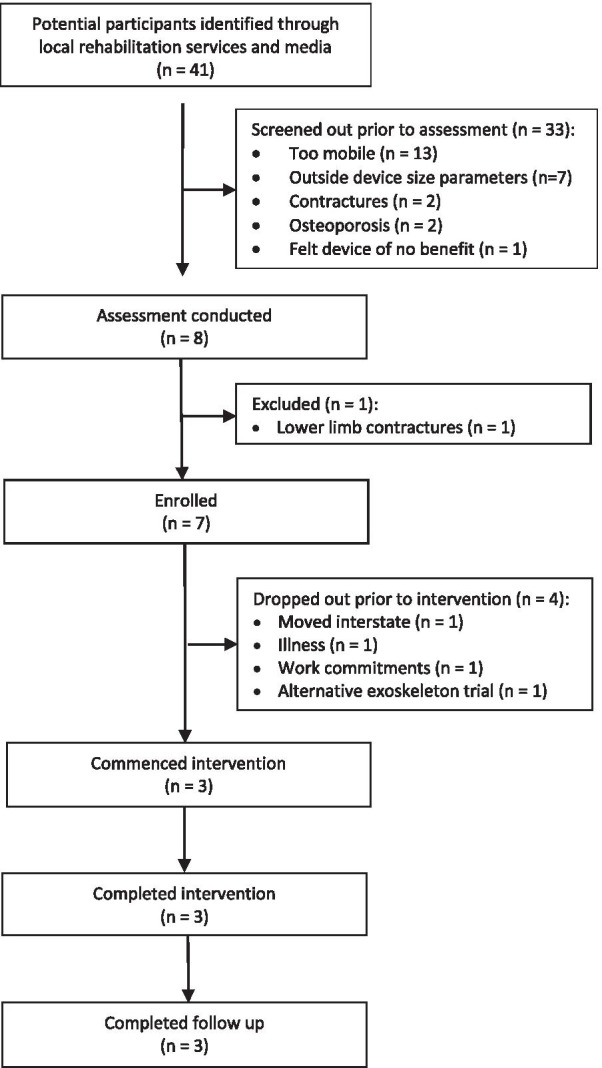
Flow of participants through the study

Recruitment occurred from October 2018 to July 2019. Forty-one potential participants were considered for the research, with seven deemed eligible (Fig. 1). The two most common reasons for people being ineligible to participate were that they were too mobile or could not fit within the device parameters for weight and height (Fig. 1). Three participants completed the full duration of the trial, in a median of 15 weeks (range 12–18). Interruptions occurred due to participant illness and other clinical appointments. There were no adverse events.

Participant one (P1) was a 25-year-old female who suffered a traumatic SCI at level C5, classification ASIA A. She transferred using a hoist and was fully dependent for all care. She was engaged in external physiotherapy services two sessions per week, which remained the same for the duration of the study.

Participant two (P2) was a 53-year-old female who suffered an ASIA C non-traumatic SCI at L3. She could pivot transfer independently and was independently ambulant very short distances within her home, with a forearm support frame. She could not push up into standing and relied on pulling up on her forearm support frame, thus limiting independence in mobility. She therefore predominantly used a powered wheelchair for mobility. She had a comprehensive clinic based (2 days a week) and home program (4 days a week) of physiotherapy throughout the duration of the study.

Participant three (P3) was a 30-year-old male who sustained a traumatic C6, ASIA B SCI. Upon enrolment, he required assistance for all transfers, mostly with a hoist, but completed car transfers with a slide board and assistance of one. He also used a powered wheelchair for mobility. P3 was engaged in physiotherapy strength and cardiovascular exercise on enrolment, which increased in frequency from one to two sessions per week by the commencement of the intervention, and then remained the same.

Participants completed similar exercises, as available in the REX, including squats, leg swings, lunges, side steps, and walking forwards and backwards. Upper limb exercises were used to encourage trunk activity. The range of exercises remained the same throughout the study. The only change was in the duration of session for P1, who in the first six sessions was unable to tolerate more than 15 min in standing due to autonomic dysreflexia. However, by the 11th session, she was able to tolerate moving forwards 8 steps. Her standing tolerance increased to 30 min by the mid-intervention assessment. From session one, P2 and P3 could tolerate 30 min in standing.

Transferring P1 into the device was challenging initially, as she was unable to assist with positioning her pelvis far enough into the device to be harnessed in. This was overcome with the use of a slide sheet and the assistance of two people.

### Primary outcome measure: function

**Fig. 2:**
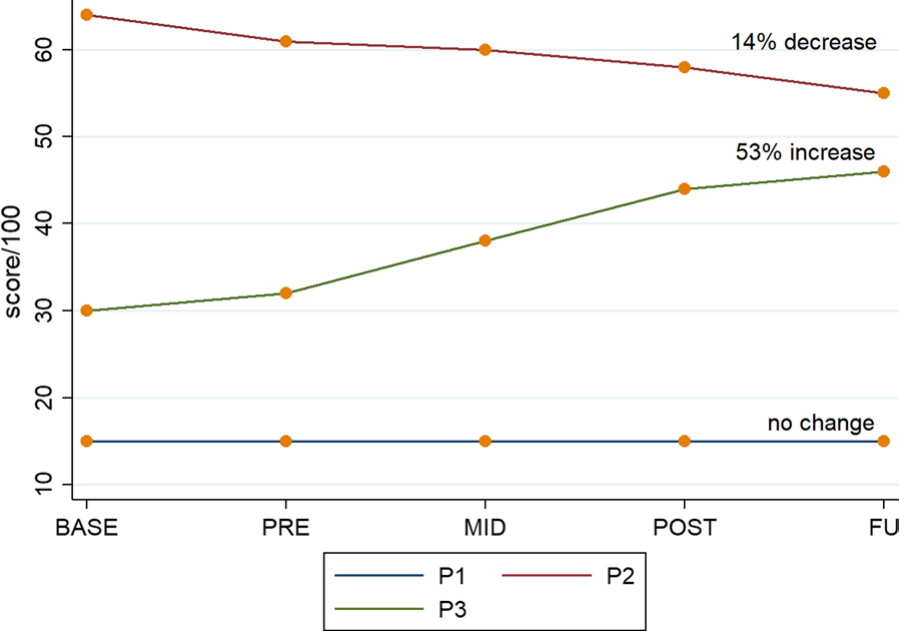
Function: Results for SCIM-III (*n* = *3*)*; annotation indicates change between base and follow up*

There was variability in baseline SCIM-III scores, symptom stability, and overall change over time across participants, with one improving, one not changing, and one deteriorating (Fig. 2).

### Other physical outcomes

**Fig. 3:**
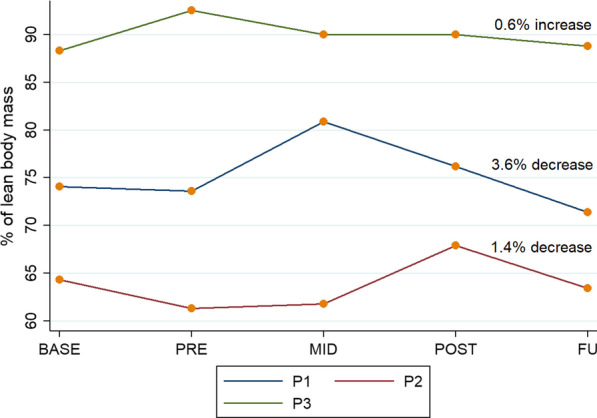
Body Composition: Results for lean body mass across all time points (*n* = *3*)*; annotation indicates change between base and follow up*

Clinical stability was not demonstrated during the wait phase of the study, with changes in some measures for some participants between week 0 and week 12. None of the participants were able to complete either the FR or FTSST at any point during the study. Spasticity was not present in any of the three muscle groups assessed in any participant at any time point. There was no overall change in the LEMS for P1 or P3. P2 showed pre-post intervention improvement of 4/50 in the LEMS, with the change occurring in the second half of the intervention phase. She also had an improvement in both grip and quads strength. The improvement in the LEMS and quads strength were not maintained at follow up. P1 and P2 both showed pre-post intervention improvement in lean body mass, compared with a worsening during the wait and follow up phases. P3 showed no overall change from enrolment to follow up. (Table 1; Fig. 3).

**Table 1:** Results from physical outcome measures

Physical outcome measures	Enrolment week 0	Baseline week 12	Mid-intervention week 18	Post-intervention week 24	Follow up week 36
LEMS (/50)					
Participant 1	0	0	0	0	0
Participant 2	20	21	21	25	23
Participant 3	1	4	2	2	1
Grip strength^a^					
Participant 2	19	20.5	22	23	23
Quads strength L^a^					
Participant 2	17	19	18	21	17

LEMS, Lower extremity motor score

^a^Participants 1 and 3 could not complete strength testing

### Other health-related outcomes

Changes in fatigue, health related QoL and mood were inconsistent between and within P1 and P2. Trends towards improvement over time in all three outcomes were evident for P3 (Table 2).

**Table 2:** Results for other health related outcomes

Outcome measures	Enrolment week 0	Baseline week 12	Mid-intervention week 18	Post-intervention week 24	Follow up week 36
FAS (/50)					
Participant 1	14	13	12	12	19
Participant 2	18	12	12	13	12
Participant 3	31	29	26	23	21
SF-8 (/42)					
Participant 1	18	11	12	11	23
Participant 2	9	11	14	9	12
Participant 3	33	34	28	26	24
HADS (/42)					
Participant 1	5	0	5	0	7
Participant 2	1	1	2	1	4
Participant 3	24	18	16	14	11

FAS, Fatigue assessment scale; SF-8, Short-form 8 health related quality of life questionnaire; HADS, Hospital anxiety and depression scale

### Participants’ perceptions of robotic therapy

**Fig. 4:**
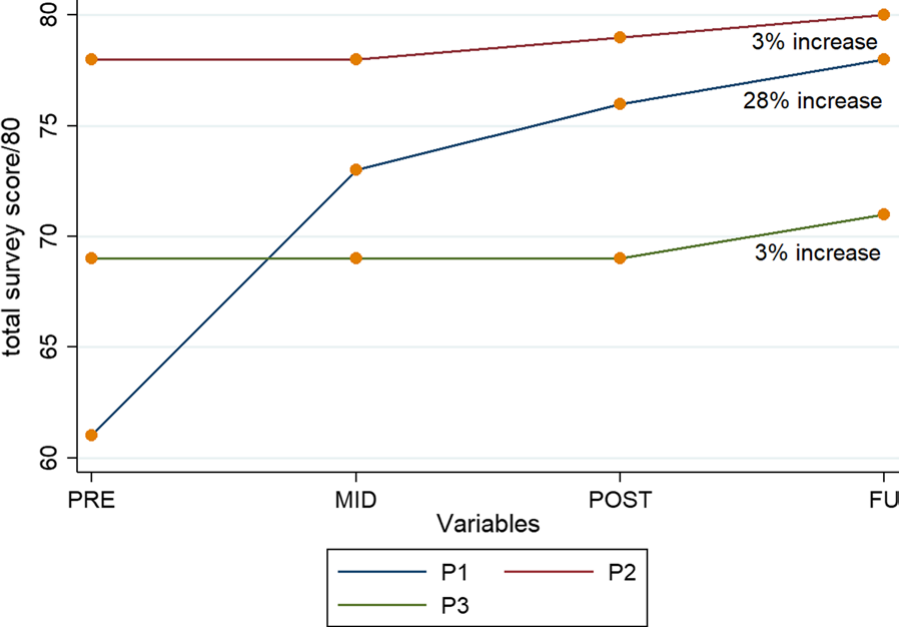
Perceptions of Therapy: Total scores for closed survey questions (*n* = *3*)*; annotation indicates change between base and follow up*

Over the course of the study, P1 gave increasing scores with regards to perceived safety, comfort and useability. Responses for the other two participants showed minimal change during the study. All three participants indicated a desire to continue participating with a score of 5/5 for that domain at all timepoints. Negative responses regarding use of the device were that it “moves slowly” and is “big”. Positive comments included being able to “look people in the eye”, “doing exercises I can’t in normal physio”, “standing upright and straight” and “feeling a stretch and tingling in my legs”. The overall scores were inconsistent between participants and timepoints, but over 60/80 throughout. See Fig. 4 for closed question survey results.

## Discussion

This study has demonstrated that it is safe and practical to deliver a program of 12 weeks of weightbearing exercise in a free-standing lower limb robotic exoskeleton in those with severe mobility impairment after SCI. The intervention was also deemed acceptable to participants, with this perception improving with increased familiarity with the device, and all participants completing all 24 intervention sessions. Whilst retention was high once intervention commenced, the recruitment rate was low, with only three participants of a possible 41 being eligible and able to participate. Initial complexities with transferring in and out of the device were overcome within two sessions, and tolerance of the upright position improved for P1, who had a history of frequently experiencing autonomic dysreflexia. Whilst the sample size was small, there were positive trends shown in some outcome measures, particularly for P3, who had a high-level incomplete SCI. We did not observe consistent trends in physical and other health related outcomes in this sample, which could be because of individual responses to therapy, the sensitivity of the tools, or confounders such as changed behaviour or activity outside of the trial.

Participant three, with sensory incomplete tetraplegia, made functional gains throughout the study. By the end of the study, his functional ability had improved to the extent that he no longer required a hoist for any transfers. Additionally, P1 gained tolerance of being in standing without suffering repeated episodes of autonomic dysreflexia and could therefore exercise in weightbearing for longer periods. These findings support the suggestion by Fritz et al. [9] that even those with complete high cervical injuries may benefit from the experience of being upright and having their postural muscles challenged. The strength gains for P2 did not translate to functional gains, as she scored lower over time on the SCIM-III due to her decision to use her powered wheelchair more at home for convenience, rather than walking with the walking frame. Anecdotally, P1 reported improved trunk control and sense of safety during sling hoist transfers during the study. This was apparent in the intervention sessions, as she required maximum assistance initially to maintain upright posture in the hoist sling when transferring into the device, but the assistance needed was significantly reduced by the end of the study. These reported benefits were not reflected in any of our outcome measures, as her SCIM score remained the same throughout the study. Whilst a minimum clinically important difference has been difficult to establish for those with SCI [45], the 53% improvement in SCIM scores observed for P3, and concurrent improvements in level of independence, are arguably worth further investigation with larger scale trials, with a more heterogenous sample of participants, to determine what aspects of the therapy and what individual characteristics of the participant led to this favourable outcome. This would enable therapy to be targeted to those individuals most likely to benefit from this type of therapy in the future.

There were positive changes in body composition during the study. Although from enrolment to follow up, two of the three participants had a decrease in their lean body mass, the same two showed improvements in their lean body mass during the intervention phase of the study (2.6% and 6.6%), which may reflect increased activity, particularly as one of the participants did not exercise regularly outside of the study due to the level and severity of her injury, and the other completed all her exercise outside of the study in sitting. Improvement in lean body mass has many health benefits [46]. Previous research, using a device requiring upper limb support, demonstrated that 52 weeks of training yielded positive changes to body composition [6]. A 2019 study found that for every percentage increase in lean body mass, there is a corresponding increase of 9% in the SCIM for tetraplegic patients [47]. Whilst our results do not reflect this correlation, further research with a larger sample is needed, as the positive changes we found over a shorter period, in a more supportive exoskeleton, may support clinical application.

One participant (P3) had improvements in fatigue, QoL, and mood, however, results for other participants were inconsistent. The same participant experienced the greatest change in level of function. We postulate that this may be due to this participant having a high level, but incomplete injury, characteristics which may lend themselves to therapy in such a supportive device. The improvement in function seen for this participant across the study may correlate with the improvements in other health related measures, a finding which needs further exploration.

One reliable finding from this study was acceptability of therapy facilitated by the exoskeleton to those who enrolled. This supports the general positive attitude towards technology in rehabilitation found by other authors [48, 49]. All participants reported that that they had “nothing to lose” by participating and wanted to be able to experience something different to routine physiotherapy. The increase of 28% from pre-intervention to follow up for P1, may be explained by her severe injury, and regular experience of autonomic dysreflexia in early sessions. The two domains which changed over the course of the study were ‘perceived safety’ and ‘comfort’. The increased scores in these domains may indicate higher confidence in, and acceptance of therapy with this device once she became more familiar with it. A 2019 study into user perspectives on walking with an exoskeleton found that the sense of self, engagement and motivation seemed to be strong upon standing [50]. Other research into the perspectives of end users of robotic devices has found that this mode of therapy did not meet expectations in terms of the perceived benefit [51]. Whilst we did not specifically investigate these areas, the continued desire to use the device beyond the study suggests that participants perceived benefit from the therapy. Comfort and useability were identified as high priorities in a 2018 study by Lajeunesse et al. [28], and both of these criteria scored highly in our study. The overall positive responses, and completion of all the intervention sessions further supports the acceptability of the intervention. We concur with other researchers who have concluded that future research and development needs to ensure these devices have maximum capacity for, and appeal to, the intended end users [9, 28, 52, 53]. Although acceptability of the intervention was high, it must be noted that one eligible participant, with a lower-level injury, chose not to enroll in the study, after being offered to trial the ReWalk exoskeleton. This implies greater perceived benefit with an alternative device, for that person.

This study included a wide range of outcome measures to analyse not just potential functional and physical benefits, but also fatigue, QoL, mood, and perceptions of the therapy itself, to provide a comprehensive evaluation. Contrary to previous research this study did not focus on gait parameters, as the target users of this type of device are non-ambulators. However, very low eligibility based on the required criteria for size and safety lead to a small sample size, and the clinical stability of the population was also not clear, as there were changes in some outcome measures during the wait phase. These factors make interpretation of the results difficult, and inferences to the population, and statistical analyses were not possible. Whilst those who used the device were positive about it, they are three out of the 41 potential participants identified during the recruitment phase, which is a small and potentially unrepresentative sample of the local SCI population. A further limitation of this study was the lack of a detailed record of the activities which participants were engaged in outside the study, and future research should consider accurate documentation of this to ensure any potential confounders are accounted for. It would also be pertinent to incorporate the analysis of the potential cardiovascular benefits of this type of therapy, in those with SCI, to compliment the analysis of lean body mass, and to compare the findings with previous research on healthy, MS and stroke participants [54].

## Conclusions

This study has shown that a 12-week intervention program using a free-standing exoskeleton for weightbearing exercise in those with severe mobility impairment, as a result of SCI, is acceptable, safe and achievable, within the context of delivering an intervention. However, there are significant limitations to feasibility in terms of the potential scale of recruitment, and a qualitative evaluation of the potential barriers to this type of therapy is recommended. This very preliminary evidence is encouraging particularly for those with incomplete high-level tetraplegia, across several health domains. However, on select outcomes all those with severe mobility impairment demonstrated positive results. These findings require further investigation with a larger sample to fully determine the potential for free-standing exoskeletal devices to have clinical application in those with severe mobility impairment post SCI.

## Supplementary Information

**Additional file 1: Appendix S1.** Survey.

## Data Availability

The datasets used and analysed during the current study are available from the corresponding author upon reasonable request.

## Abbreviations

SCI: Spinal cord injury
ASIA: American spinal cord injury association
SCIM-III: Spinal cord independence measure
MoCA: Montreal cognitive assessment
LEMS: Lower extremity motor score
FTSST: Five times sit to stand test
FR: Functional reach
BIA: Bioelectrical impedance analysis
HADS: Hospital anxiety and depression scale
FAS: Fatigue assessment scale
QoL: Quality of life
P1: Participant one
P2: Participant two
P3: Participant three
