# Supplementary material for: Physiotherapy using a free-standing robotic exoskeleton for patients with spinal cord injury: a feasibility study
Source: J Neuroeng Rehabil. 2021 Dec 25;18:180. doi: 10.1186/s12984-021-00967-4 (PMC8709973; doi:10.1186/s12984-021-00967-4)
Supplement: Supplementary file 1 — Additional file 1: Appendix S1. Survey. [file 12984_2021_967_MOESM1_ESM.docx]

**Participant Perception of Therapy using HELLEN**

**Perceived Safety**

Please rate your emotions about using HELLEN

Anxious 1 2 3 4 5 Relaxed

Agitated 1 2 3 4 5 Calm

Pessimistic 1 2 3 4 5 Optimistic **TOTAL /15 =**

**Likeability**

Please rate your impression of HELLEN

Dislike 1 2 3 4 5 Like

Awful 1 2 3 4 5 Pleasant

Discouraging 1 2 3 4 5 Motivating

Depressing 1 2 3 4 5 Confidence building **TOTAL /20 =**

**Comfort**

Please rate your impression of HELLEN

Constricted 1 2 3 4 5 Free

Uncomfortable 1 2 3 4 5 Comfortable

Cumbersome 1 2 3 4 5 Manageable

Painful 1 2 3 4 5 Pain Free

Exhausting 1 2 3 4 5 Invigorating **TOTAL /25 =**

**Useability**

Please rate your impression of HELLEN

Complex to adjust 1 2 3 4 5 Easy to adjust

Of no benefit 1 2 3 4 5 Beneficial

Time intensive 1 2 3 4 5 Quick **TOTAL /15 =**

**Given the opportunity, would you like to continue receiving therapy in HELLEN?**

Definitely not 1 2 3 4 5 Definitely **TOTAL /5 =**

**TOTAL /80 =**

Please tell us what you **liked** about using HELLEN to assist your therapy:

Please tell us what you **disliked** about using HELLEN to assist your therapy:
